# HLA-B allele and haplotype diversity among Thai patients identified by PCR-SSOP: evidence for high risk of drug-induced hypersensitivity

**DOI:** 10.3389/fgene.2014.00478

**Published:** 2015-01-22

**Authors:** Apichaya Puangpetch, Napatrupron Koomdee, Montri Chamnanphol, Thawinee Jantararoungtong, Siwalee Santon, Santirhat Prommas, Yaowaluck Hongkaew, Chonlaphat Sukasem

**Affiliations:** ^1^Division of Pharmacogenomics and Personalized Medicine, Department of Pathology, Faculty of Medicine Ramathibodi Hospital, Mahidol UniversityBangkok, Thailand; ^2^Laboratory for Pharmacogenomics, Somdech Phra Debaratana Medical Center, Ramathibodi Hospital, Mahidol UniversityBangkok, Thailand

**Keywords:** *HLA-B*, pharmacogenomic markers, Thai, PCR-SSOP, SCARs, drug hypersensitivity

## Abstract

**Background:** There are 3 classes of *HLA* molecules; *HLA* class I, II and III, of which different classes have different functions. *HLA-B* gene which belongs to *HLA* class I play an important role predicting drug hypersensitivity.

**Materials and Methods:** Nine hundred and eighty-six Thai subjects who registered at a pharmacogenomics laboratory were determined for *HLA-B* genotype using a two-stage sequence-specific oligonucleotide probe system (PCR-SSOP).

**Results:** In this study, *HLA-B* alleles did not deviate from Hardy-Weinberg equilibrium (*P* > 0.05). The most common *HLA-B* alleles observed in this population were HLA-B^*^46:01 (11.51%), HLA-B^*^58:01 (8.62%), HLA-B^*^40:01 (8.22%), HLA-B^*^15:02 (8.16%) and HLA-B^*^13:01 (6.95%). This finding revealed that *HLA-B* allele frequency in the Thai population was consistent with the Chinese population (*p* > 0.05), however, differed from the Malaysian population (*p* < 0.05). The top five *HLA-B* genotypes were HLA-B^*^40:01/46:01 (2.13%), HLA-B^*^46:01/46:01 (2.03%), HLA-B^*^40:01/58:01 (2.03%), HLA-B^*^46:01/58:01 (1.93%) and HLA-B^*^15:02/46:01 (1.83%). This study found that 15.92% of Thai subjects carry HLA-B^*^15:02, which has been associated with carbamazepine-induced severe cutaneous adverse drug reactions (SCARs). Moreover, 16.33% of Thai subjects carry the HLA-B^*^58:01 allele, which has been associated with allopurinol-induced SCARs.

**Conclusion:** This study demonstrates a high diversity of *HLA-B* polymorphisms in this Thai population. The high frequency of *HLA-B* pharmacogenomic markers in the population emphasizes the importance of such screening to predict/avoid drug hypersensitivity.

## Introduction

*Human leukocyte antigen (HLA)* genes are located in the short arm of chromosome 6, are part of a large family of genes, and are inherited from both parents, one of paternal origin and one of maternal origin for each of the loci (Alper et al., 2006). HLA proteins are present at the cellular surface of antigen presenting cells. There are 3 classes of HLA. Class I loci (HLA-A, -B, -C) present endogenous antigens such as viral protein or tumor antigen to CD8^+^ T cells. Class II loci (HLA-DR, -DQ, -DP) present exogenous antigens to CD4^+^ T cells. Class III (Bf, C2, C4A) are involved in the complement system. *HLA* genes are highly polymorphic, which becomes important in associations of susceptibility or resistance to disease, development of tumor processes, molecular vaccine development (Dawson et al., 2001) and determination of organ or tissue transplant survival (Dhaliwal et al., 2003; Avila-Rios et al., 2009). In addition, *HLA* allele frequency might be useful in anthropological studies. Previous studies have reported *HLA* allele and haplotype frequencies of Thai populations. The most common *HLA* haplotypes were A^*^33-Cw^*^0302-B^*^5801-DRB^*^0301-DQB1^*^02 (4.6%), A^*^0207-Cw^*^01-B^*^4601-DRB1^*^09-DQB1^*^0303 (3.4%) and A^*^33-Cw^*^07 (01-03)-B^*^44-DRB1^*^07-DQB1^*^02 (2.6%) (Kupatawintu et al., 2010; Romphruk et al., 2010).

*HLA-B* alleles have been used as a marker for predicting drug-induced adverse reactions (Sukasem et al., 2014a) and are a major contributor to hypersensitivity reactions involving direct stimulation of immune effector cells and imitating an allergic reaction. Several studies reported that allopurinol-induced severe cutaneous adverse reactions (SCARs) are strongly associated with the HLA-B^*^58:01 allele in Han Chinese and Thais (Hung et al., 2005; Tassaneeyakul et al., 2009; Jantararoungtong et al., 2014). The HLA-B^*^15:02 allele was strongly related to carbamazepine-induced Steven-Johnson syndrome (SJS)-toxic epidermal necrolysis (TEN) in Taiwanese and Thai populations (Tassaneeyakul et al., 2010; Chen et al., 2011; Sukasem et al., 2014b). Importantly, Thais showed cutaneous adverse reactions associated with HLA-B^*^35:05 (Chantarangsu et al., 2009, 2011). In addition, the HLA-B^*^57:01 allele is involved in abacavir-induced hypersensitivity reaction (Mallal et al., 2008). Consequently, screening of *HLA-B* genotyping before prescription of medication may reduce the risk of SCARs and drug hypersensitivity reaction.

Presently, *HLA-B* genotyping is available in clinical practices before commencing therapy with the aforementioned drugs in Thailand (Sukasem et al., 2014a). This study aimed to determine the distribution of *HLA-B* haplotypes, and to establish the most common *HLA* haplotype-associated drug induced hypersensitivity using routine pharmacogenomic laboratory procedures. Over a period of 3 years, *HLA-B* allele genotype was analyzed in 986 Thai people registered at the Laboratory for Pharmacogenomics, Ramathibodi Hospital, Thailand. The frequency of *HLA-B* alleles and *HLA-B* genotypes were determined and used to define the probability associated with a Pharmacogenomic marker indicating adverse drug reaction.

## Materials and methods

### Subjects and characteristics

A descriptive, observational, cross-sectional, retrospective study was conducted in 986 unrelated individuals. Data were collected at the Laboratory for Pharmacogenomics, Somdech Phra Debaratana Medical Center (SDMC), Ramathibodi Hospital, Bangkok, Thailand during August 2011–June 2014. Among these patients, there were 590 (59.83%) males and 396 (40.17%) females. The mean age of patients at recruitment was 41.98 ± 4.40 years. The study was approved by the Ethics Committee of the Faculty of Medicine, Ramathibodi Hospital, Mahidol University. The protocol number is ID 04-56-24.

### Genomic DNA extraction

Blood samples were collected into EDTA tubes. DNA was isolated using the MagNA Pure automated extraction system (Roche diagnostics, USA), which uses magnetic-bead technology with a lysis buffer and proteinase K. Nucleic acids bind to the surface of the magnetic glass particles. Cellular debris was removed by several washing steps and the purified nucleic acids were eluted. From the 1 ml input volume of EDTA-whole blood, 200 μL output volume of extracted genomic DNA product was obtained.

The quality of genomic DNA was assessed by using Nano Drop ND-1000. Genomic DNA was detected by measuring absorbance at 260 nm. Purity of the sample was evaluated by calculation of the optical density (OD) ratio, 260/280 nm. The recommended purified genomic DNA template for this study was 20 ng, and the OD ratio greater than 1.7. All DNA was aliquotted and stored at −20°C before analysis.

### *HLA-B* typing

*HLA-B* genotyping was carried out using the Luminex™ Multiplex Technology (Luminex^®^IS 100, USA) based on Polymerase Chain Reaction-sequence specific oligonucleotides probe (PCR-SSOP) principles. Briefly, the PCR products were hybridized against a panel of oligonucleotide probes coated onto polystyrene microspheres. Probe sequences were complementary to stretches of polymorphic sequence within the target *HLA-B* alleles. The amplicon-probe complex was visualized using a colorimetric reaction and fluorescence detection technology. Data analyses for the *HLA-B* assays were performed with HLA fusion™2.0 software.

### Statistical analysis

The *HLA-B* allele frequencies of the samples were assayed by direct counting and, subsequently, by dividing the total number of occurrences of that allele by the total number of alleles at that locus in the population. The samples were also evaluated for Hardy-Weinberg equilibrium using the Chi-square or Fisher's exact test if the number in any cell of the 2 × 2 contingency tables was less than five.

## Results

### *HLA-B* allele frequency

The study population comprised 986 samples which were obtained from Laboratory for Pharmacogenomics, Somdech Phra Debaratana Medical Center (SDMC), Ramathibodi Hospital, Thailand. The frequency of *HLA-B* alleles in 986 Thai patients is shown in Table 1. Overall, 116 different *HLA-B* alleles were identified in this study, and HLA-B^*^46:01 (11.51%) was the predominant allele in this population. The most frequent alleles were HLA-B^*^46:01 (11.51%), HLA-B^*^58:01 (8.62%), HLA-B^*^40:01 (8.22%), HLA-B^*^15:02 (8.16%), HLA-B^*^13:01 (6.95%) and HLA-B^*^44:03 (4.21%). The frequencies of *HLA-B* alleles do not significantly deviate from Hardy-Weinberg equilibrium.

**Table 1:** **Identified *HLA-B* allelic frequencies in Thai population (n = 986)**.

**Alleles**	**No. of alleles**	**AF (%)**	**Estimated genotype**	**No. of genotypes**	**Genotype frequency (%)**	**HW *p*-value**
B^*^07:02	15	0.76	0.06	0	0.00	1
B^*^07:05	38	1.93	0.37	0	0.00	1
B^*^07:13	1	0.05	0.00	0	0.00	1
B^*^07:14	1	0.05	0.00	0	0.00	1
B^*^08:01	13	0.66	0.04	0	0.00	1
B^*^08:02	1	0.05	0.00	0	0.00	1
B^*^08:03	1	0.05	0.00	0	0.00	1
B^*^08:12	1	0.05	0.00	0	0.00	1
B^*^13:01	*137*	6.95	4.76	6	0.61	1
B^*^13:02	27	1.37	0.18	0	0.00	1
B^*^13:03	1	0.05	0.00	0	0.00	1
B^*^13:39	3	0.15	0.00	0	0.00	1
B^*^14:13	1	0.05	0.00	0	0.00	1
B^*^15:01	27	1.37	0.18	1	0.10	1
B^*^15:02	161	8.16	6.57	8	0.81	1
B^*^15:04	4	0.20	0.00	0	0.00	1
B^*^15:06	1	0.05	0.00	0	0.00	1
B^*^15:07	3	0.15	0.00	0	0.00	1
B^*^15:11	5	0.25	0.01	0	0.00	1
B^*^15:12	12	0.61	0.04	0	0.00	1
B^*^15:13	11	0.56	0.03	0	0.00	1
B^*^15:17	4	0.20	0.00	0	0.00	1
B^*^15:18	3	0.15	0.00	0	0.00	1
B^*^15:20	2	0.10	0.00	0	0.00	1
B^*^15:21	3	0.15	0.00	0	0.00	1
B^*^15:22	1	0.05	0.00	0	0.00	1
B^*^15:25	28	1.42	0.20	0	0.00	1
B^*^15:27	2	0.10	0.00	0	0.00	1
B^*^15:31	4	0.20	0.00	0	0.00	1
B^*^15:32	7	0.35	0.01	0	0.00	1
B^*^15:35	17	0.86	0.07	1	0.10	1
B^*^15:88	1	0.05	0.00	0	0.00	1
B^*^18:01	63	3.19	1.01	0	0.00	1
B^*^18:01	1	0.05	0.00	0	0.00	1
B^*^18:02	45	2.28	0.51	0	0.00	1
B^*^18:02	1	0.05	0.00	0	0.00	1
B^*^18:09	1	0.05	0.00	0	0.00	1
B^*^18:18	1	0.05	0.00	0	0.00	1
B^*^27:03	3	0.15	0.00	0	0.00	1
B^*^27:04	32	1.62	0.26	0	0.00	1
B^*^27:06	18	0.91	0.08	0	0.00	1
B^*^27:61	10	0.51	0.03	0	0.00	1
B^*^27:86	1	0.05	0.00	0	0.00	1
B^*^35:01	29	1.47	0.21	0	0.00	1
B^*^35:02	2	0.10	0.00	0	0.00	1
B^*^35:03	17	0.86	0.07	0	0.00	1
B^*^35:05	40	2.03	0.41	1	0.10	1
B^*^35:08	1	0.05	0.00	0	0.00	1
B^*^35:11	1	0.05	0.00	0	0.00	1
B^*^35:13	1	0.05	0.00	0	0.00	1
B^*^35:20	1	0.05	0.00	0	0.00	1
B^*^35:23	1	0.05	0.00	0	0.00	1
B^*^35:58	1	0.05	0.00	0	0.00	1
B^*^35:68	2	0.10	0.00	0	0.00	1
B^*^37:01	8	0.41	0.02	0	0.00	1
B^*^38:01	2	0.10	0.00	0	0.00	1
B^*^38:02	61	3.09	0.94	3	0.30	0.624
B^*^38:17	1	0.05	0.00	0	0.00	1
B^*^38:22	1	0.05	0.00	0	0.00	1
B^*^38:23	2	0.10	0.00	0	0.00	1
B^*^39:01	14	0.71	0.05	0	0.00	1
B^*^39:09	18	0.91	0.08	0	0.00	1
B^*^39:15	13	0.66	0.04	0	0.00	1
B^*^39:24	5	0.25	0.01	0	0.00	1
B^*^40:01	162	8.22	6.65	6	0.61	1
B^*^40:02	30	1.52	0.23	0	0.00	1
B^*^40:03	1	0.05	0.00	0	0.00	1
B^*^40:04	7	0.35	0.01	0	0.00	1
B^*^40:06	14	0.71	0.05	1	0.10	1
B^*^40:09	1	0.05	0.00	0	0.00	1
B^*^40:10	6	0.30	0.01	0	0.00	1
B^*^40:23	1	0.05	0.00	0	0.00	1
B^*^40:59	1	0.05	0.00	0	0.00	1
B^*^41:01	1	0.05	0.00	0	0.00	1
B^*^41:10	1	0.05	0.00	0	0.00	1
B^*^44:01	2	0.10	0.00	0	0.00	1
B^*^44:02	14	0.71	0.05	0	0.00	1
B^*^44:02	1	0.05	0.00	0	0.00	1
B^*^44:03	83	4.21	1.75	1	0.10	1
B^*^44:54	1	0.05	0.00	0	0.00	1
B^*^46:01	227	11.51	13.07	20	2.03	0.292
B^*^46:12	26	1.32	0.17	1	0.10	1
B^*^46:16	1	0.05	0.00	0	0.00	1
B^*^48:01	9	0.46	0.02	0	0.00	1
B^*^48:03	7	0.35	0.01	0	0.00	1
B^*^48:21	1	0.05	0.00	0	0.00	1
B^*^50:01	11	0.56	0.03	0	0.00	1
B^*^51:01	65	3.30	1.07	0	0.00	1
B^*^51:02	16	0.81	0.06	0	0.00	1
B^*^51:04	4	0.20	0.00	0	0.00	1
B^*^51:06	3	0.15	0.00	0	0.00	1
B^*^51:07	1	0.05	0.00	0	0.00	1
B^*^51:43	1	0.05	0.00	0	0.00	1
B^*^51:45	1	0.05	0.00	0	0.00	1
B^*^52:01	43	2.18	0.47	0	0.00	1
B^*^52:07	1	0.05	0.00	0	0.00	1
B^*^53:17	5	0.25	0.01	0	0.00	1
B^*^54:01	21	1.06	0.11	0	0.00	1
B^*^54:01	2	0.10	0.00	0	0.00	1
B^*^54:04	1	0.05	0.00	0	0.00	1
B^*^54:16	1	0.05	0.00	0	0.00	1
B^*^55:01	8	0.41	0.02	0	0.00	1
B^*^55:02	23	1.17	0.13	0	0.00	1
B^*^55:03	1	0.05	0.00	0	0.00	1
B^*^55:04	2	0.10	0.00	0	0.00	1
B^*^55:10	1	0.05	0.00	0	0.00	1
B^*^55:44	2	0.10	0.00	0	0.00	1
B^*^56:01	15	0.76	0.06	0	0.00	1
B^*^56:01	1	0.05	0.00	0	0.00	1
B^*^56:02	1	0.05	0.00	0	0.00	1
B^*^56:04	6	0.30	0.01	0	0.00	1
B^*^56:16	3	0.15	0.00	0	0.00	1
B^*^57:01	30	1.52	0.23	0	0.00	1
B^*^57:21	2	0.10	0.00	0	0.00	1
B^*^58:01	170	8.62	7.33	9	0.91	1
B^*^58:34	1	0.05	0.00	0	0.00	1
Total	1972	100				

AF, Allele frequencies; H-W, Hardy-Weinberg equilibrium.

### Frequency of *HLA-B* genotype occurrence

Overall, there were 448 different *HLA-B* genotypes in the 986 Thai individuals analyzed. The most frequently observed HLA-B genotype was HLA-B^*^40:01/46:01. The 10 most common genotype frequencies in this Thai population were HLA-B^*^40:01/46:01 (2.13%), *HLA*-B^*^46:01/46:01 (2.03%), HLA-B^*^40:01/58:01 (2.03%), HLA-B^*^46:01/58:01 (1.93%), HLA-B^*^15:02/46:01 (1.83%), HLA-B^*^15:02/40:01 (1.52%), HLA-B^*^13:01/58:01 (1.32%), HLA-B^*^13:01/15:02 (1.22%), HLA-B^*^13:01/46:01 (1.12%), HLA-B^*^38:02/46:01 (1.12%), HLA-B^*^15:02/44:03 (1.12%), and HLA-B^*^13:01/40:01 (1.01%). None of these genotypes were present in more than 5% of the population. Table 2 shows the frequency of the top 10 genotypes the Thai population in this study.

**Table 2:** **Top 10 common genotypes in the Thais (**n** = 986)**.

**Genotypes**	**No. of subjects**	**Frequency (%)**
B^*^40:01/*46:01*	21	2.13
B^*^46:01/*46:01*	20	2.03
B^*^40:01/*58:01*	20	2.03
B^*^46:01/*58:01*	19	1.93
B^*^15:02/*46:01*	18	1.83
B^*^15:02/*40:01*	15	1.52
B^*^13:01/*58:01*	13	1.32
B^*^13:01/*15:02*	12	1.22
B^*^13:01/*46:01*	11	1.12
B^*^38:02/*46:01*	11	1.12
B^*^15:02/*44:03*	11	1.12
B^*^13:01/*40:01*	10	1.01

### Frequency of the strong prediction of *HLA-B* pharmacogenomics markers in thai population

With regard to *HLA-B* pharmacogenomic markers in the Thai population, high frequencies of HLA-B^*^ 58:01 (16.33%) were observed, followed by HLA-B^*^15:02 (15.92%), HLA-B^*^ 35:05 (4.36%), and HLA-B^*^57:01 (3.04%). Table 3 shows the frequencies of the genotypes of *HLA-B* pharmacogenomic markers in this Thai population. Of the 986 subjects who were included in the study, 161 subjects (16.43%) were found to carry the HLA-B^*^58:01 allele. One hundred and fifty-two subjects carry the heterozygous HLA-B^*^58:01 genotype and 9 subjects carry the homozygous HLA-B^*^58:01 genotype. Among these, 56.02% were males and 43.98% were female.

**Table 3:** **Pharmacogenomics markers of HLA-B genotype in a Thai population (n = 986)**.

HLA-B^*^15:02			HLA-B^*^35:05			HLA-B^*^57:01			HLA-B^*^58:01		
**Genotype**	**Subjects (n = 157)**	**%**	**Genotype**	**Subjects (n = 43)**	**%**	**Genotype**	**Subjects (n = 30)**	**%**	**Genotype**	**Subjects (n = 161)**	**%**
B^*^15:02/46:01	18	1.83	B^*^35:05/46:01	7	0.71	B^*^57:01/38:02	3	0.30	B^*^58:01/40:01	20	2.03
B^*^15:02/40:01	15	1.52	B^*^35:05/15:02	5	0.51	B^*^57:01/44:03	4	0.41	B^*^58:01/46:01	19	1.93
B^*^15:02/13:01	12	1.22	B^*^35:05/40:01	5	0.51	B^*^57:01/46:01	3	0.30	B^*^58:01/13:01	13	1.32
B^*^15:02/44:03	11	1.12	B^*^35:05/18:01	3	0.30	B^*^57:01/13:01	2	0.20	B^*^58:01/15:02	9	0.91
B^*^15:02/58:01	9	0.91	B^*^35:05/44:03	3	0.30	B^*^57:01/18:01	2	0.20	B^*^58:01/18:02	7	0.71
B^*^15:02/15:02	8	0.81	B^*^35:05/13:01	2	0.20	B^*^57:01/40:01	2	0.20	B^*^58:01/58:01	9	0.91
B^*^15:02/18:01	6	0.61	B^*^35:05/40:06	2	0.20	B^*^57:01/07:05	1	0.10	B^*^58:01/52:01	6	0.61
B^*^15:02/27:04	6	0.61	B^*^35:05/51:01	2	0.20	B^*^57:01/13:02	1	0.10	B^*^58:01/18:01	5	0.51
B^*^15:02/52:01	6	0.61	B^*^35:05/15:11	1	0.10	B^*^57:01/15:01	1	0.10	B^*^58:01/44:03	5	0.51
B^*^15:02/15:35	6	0.61	B^*^35:05/15:17	1	0.10	B^*^57:01/15:02	1	0.10	B^*^58:01/46:12	5	0.51
B^*^15:02/35:05	5	0.51	B^*^35:05/15:25	1	0.10	B^*^57:01/15:17	1	0.10	B^*^58:01/51:01	5	0.51
B^*^15:02/51:01	5	0.51	B^*^35:05/18:18	1	0.10	B^*^57:01/27:06	1	0.10	B^*^58:01/15:25	3	0.30
B^*^15:02/18:02	4	0.41	B^*^35:05/27:06	1	0.10	B^*^57:01/35:03	1	0.10	B^*^58:01/35:01	3	0.30
B^*^15:02/07:05	3	0.30	B^*^35:05/35:01	1	0.10	B^*^57:01/35:05	1	0.10	B^*^58:01/39:09	3	0.30
B^*^15:02/15:25	3	0.30	B^*^35:05/35:05	1	0.10	B^*^57:01/37:01	1	0.10	B^*^58:01/40:02	3	0.30
B^*^15:02/38:02	3	0.30	B^*^35:05/39:09	1	0.10	B^*^57:01/38:23	1	0.10	B^*^58:01/53:17	3	0.30
B^*^15:02/13:02	2	0.20	B^*^35:05/39:15	1	0.10	B^*^57:01/40:04	1	0.10	B^*^58:01/07:05	2	0.20
B^*^15:01/15:02	2	0.20	B^*^35:05/40:02	1	0.10	B^*^57:01/48:01	1	0.10	B^*^58:01/08:01	2	0.20
B^*^15:02/15:13	2	0.20	B^*^35:05/48:01	1	0.10	B^*^57:01/56:01	1	0.10	B^*^58:01/15:01	2	0.20
B^*^15:02/35:01	3	0.30	B^*^35:05/55:02	1	0.10	B^*^57:01/58:01	1	0.10	B^*^58:01/27:04	2	0.20
B^*^15:02/40:02	3	0.30	B^*^35:05/57:01	1	0.10				B^*^58:01/27:06	2	0.20
B^*^15:02/07:13	1	0.10	B^*^35:05/58:01	1	0.10				B^*^58:01/37:01	3	0.30
B^*^15:02/13:01	1	0.10							B^*^58:01/38:02	2	0.20
B^*^15:02/15:06	1	0.10							B^*^58:01/48:03	2	0.20
B^*^15:02/15:18	1	0.10							B^*^58:01/54:01	2	0.20
B^*^15:02/15:20	1	0.10							B^*^58:01/55:02	2	0.20
B^*^15:02/15:88	1	0.10							B^*^58:01/07:02	1	0.10
B^*^15:02/18:01	1	0.10							B^*^58:01/13:02	1	0.10
B^*^15:02/35:03	1	0.10							B^*^58:01/15:11	1	0.10
B^*^15:02/35:58	1	0.10							B^*^58:01/15:13	1	0.10
B^*^15:02/40:23	1	0.10							B^*^58:01/15:20	1	0.10
B^*^15:02/46:01	1	0.10							B^*^58:01/15:21	1	0.10
B^*^15:02/46:12	3	0.30							B^*^58:01/15:32	1	0.10
B^*^15:02/51:01	1	0.10							B^*^58:01/27:03	1	0.10
B^*^15:02/51:02	1	0.10							B^*^58:01/35:05	1	0.10
B^*^15:02/51:04	1	0.10							B^*^58:01/38:13	1	0.10
B^*^15:02/51:06	1	0.10							B^*^58:01/38:20	1	0.10
B^*^15:02/54:01	1	0.10							B^*^58:01/39:01	1	0.10
B^*^15:02/55:02	1	0.10							B^*^58:01/40:59	1	0.10
B^*^15:02/55:04	1	0.10							B^*^58:01/44:02	1	0.10
B^*^15:02/55:13	1	0.10							B^*^58:01/46:16	1	0.10
B^*^15:02/56:01	1	0.10							B^*^58:01/48:01	1	0.10
B^*^15:02/56:04	1	0.10							B^*^58:01/48:21	1	0.10
B^*^15:02/57:01	1	0.10							B^*^58:01/56:01	1	0.10
									B^*^58:01/56:04	1	0.10
									B^*^58:01/57:01	1	0.10
									B^*^58:01/07:05	1	0.10
									B^*^58:01/39:09	1	0.10

HLA-B^*^15:02 is a genetic marker previously related to induction of SJS/TEN in carbamazepine treated patients. One hundred and fifty-seven subjects (15.92%) were found to carry *HLA-B*^ ^15:02; 151 subjects (15.31%) were heterozygous for the HLA-B^*^15:02 allele and 8 subjects (0.81%) carry homozygous HLA-B^*^15:02. Males accounted for 60.23% and females 39.77%. In addition, HLA-B^*^15:02 belong to the HLA-B75 family, which consists of HLA-B^*^15:08, HLA-B^*^15:11, HLA-B^*^15:18 and HLA-B^*^15:21, and is associated with carbamazepine-induced SJS/TEN. Five subjects with HLA-B^*^15:11 heterozygous genotype and 3 subjects with HLA-B^*^15:18, HLA-B^*^15:21 heterozygous genotype were found, whereas HLA-B^*^15:08 was not found in either heterozygous and homozygous genotypes in this population.

Likewise, the HLA-B^*^3505 allele was associated with nevirapine-induced hypersensitivity reactions in HIV-infected Thai patients. Forty-three subjects (4.36%) carried the HLA-B^*^35:05 allele, one subject and 42 subjects (4.26%) carry homozygous and heterozygous HLA-B^*^35:05 genotypes, respectively, Thirty subjects (3.04%) carry heterozygous HLA-B^*^57:01, which is known to be associated with abacavir-induced hypersensitivity syndrome (AHS). No homozygous HLA-B^*^57:01 genotype was identified in this study.

## Discussion

The *HLA-B* allele frequencies of 968 Thai subjects were investigated. This is the first report of *HLA-B* genotyping in the Thai population using the Luminex HLA-SSOP method. Our data showed that the Thai population has extensive diversity at the *HLA-B* locus. One hundred and sixteen allele types were identified and the frequencies of *HLA-B* alleles did not significantly deviate from Hardy-Weinberg equilibrium. The top five alleles with frequencies over 5% include HLA-B^*^46:01 (11.51%), HLA-B^*^58:01 (8.62%), HLA-B^*^40:01 (8.22%), HLA-B^*^15:02 (8.16%), and HLA-B^*^13:01 (6.95%). These frequencies were similar to those observed in the Singaporean Chinese: HLA-B^*^40:01 (17.2%), HLA-B^*^46:01 (13.2%), HLA-B^*^58:01 (10.4%), HLA-B^*^13:01 (8.40%), HLA-B^*^15:02 (5.70%) and Hong Kong Chinese: HLA-B^*^46:01 (16.3%), HLA-B^*^40:01 (15.2%), HLA-B^*^58:01 (7.30%), HLA-B^*^15:02 (10.2%) and HLA-B^*^13:01 (7.80%) (Williams et al., 2001; Middleton et al., 2004). These data differed, however, from the Jehai population in Malaysia nearest the south of Thailand: HLA-B^*^46:01 (2%), HLA-B^*^58:01 (6%), HLA-B^*^40:01 (2%), HLA-B^*^15:02 (2%), HLA-B^*^13:01 (8%) (Jinam et al., 2010; Kupatawintu et al., 2010; Romphruk et al., 2010). This may indicate that Thais have a closer relationship with their Chinese neighbors than with Malaysia. Previous studies of *HLA* alleles in the Thai population had larger subject numbers, but reported no pharmacogenomic data, which we present in the current study. HLA-B^*^15:02 allele is found in high prevalence among people in South-East Asian countries. The frequency of the *HLA-B* allele varies among different populations suggesting that different alleles may also function in drug hypersensitivity. Currently, there has been an increase of publications associated with *HLA-B* alleles and drug hypersensitivity (Sukasem et al., 2014a).

In our study, 15.92% (*n* = 157) of Thai subjects carry HLA-B^*^15:02 genotype, which has been associated with severe adverse drug reaction in response to carbamazepine during epileptic treatment (Tassaneeyakul et al., 2010; Kulkantrakorn et al., 2012; Tangamornsuksan et al., 2013) or other aromatic amine anticonvulsants such as oxcarbazepine, phenytoin, and lamotrigine (Man et al., 2007; Hung et al., 2010; Koomdee et al., 2014). Further, the serotype HLA-B75 family, such as HLA-B^*^15:08, HLA-B^*^15:11 (0.25%, *n* = 5), HLA-B^*^15:18 (0.15%, *n* = 3) and HLA-B^*^15:21 (0.15%, *n* = 3), is associated with carbamazepine-induced SJS/TEN (Daly and Day, 2009; Lin et al., 2009). In this study, 168 subjects (17.03%) carrying members of serotype HLA-B75 family alleles may be susceptible to carbamazepine-induced SJS/TEN.

The genetic predisposition to nevirapine and abacavir-induced hypersensitivity reaction (HSR) has been reported for HLA-B^*^35:05 and HLA-B^*^57:01 alleles, respectively (Mallal et al., 2008; Chantarangsu et al., 2009, 2011). The data showed that 4.36% of Thai people carry the HLA-B^*^35:05 genotype and 3.04% carry the HLA-B^*^57:01 genotype (Chantarangsu et al., 2009). Furthermore, HLA allotypes closely related to abacavir response are HLA-B^*^57:03, HLA-B^*^57:02, and HLA-B^*^58:01 (Illing et al., 2012). Consequently, 23.73% of Thai people might be at risk of drug hypersensitivity from nevirapine or abacavir treatment.

In the present study, we found that 16.33% of Thai people carry the HLA-B^*^58:01 allele, which is associated with allopurinol hypersensitivity in gout treatment (Tassaneeyakul et al., 2009; Saokaew et al., 2014). Beside drug hypersensitivity, other studies of *HLA-B* alleles, such as HLA-B^*^46:01, report a significant increase in severity of cerebral malaria compared with patients with mild CNS symptoms (Hirayama et al., 1998). Moreover, HLA-B^*^40:01 is associated with lipodystrophy in Thai HIV-1-positive patients who received antiretroviral therapy containg stavudine (Wangsomboonsiri et al., 2010). HLA-B^*^13:01 has been suggested as a genetic marker for dermal hypersensitivity associated with Trichloroethylene, an industrial solvent (Li et al., 2007).

Our findings demonstrated the diversity of *HLA-B* alleles in the Thai population, and many more HLA associated with ADR. Other classes of HLA may be observed in upcoming studies. The subjects enrolled in the study were mostly from central Thailand. This narrow sample may restrict our calculations of *HLA-B* allele distribution in Thai populations. Future studies should consider this limitation of our survey.

**Figure 1:**
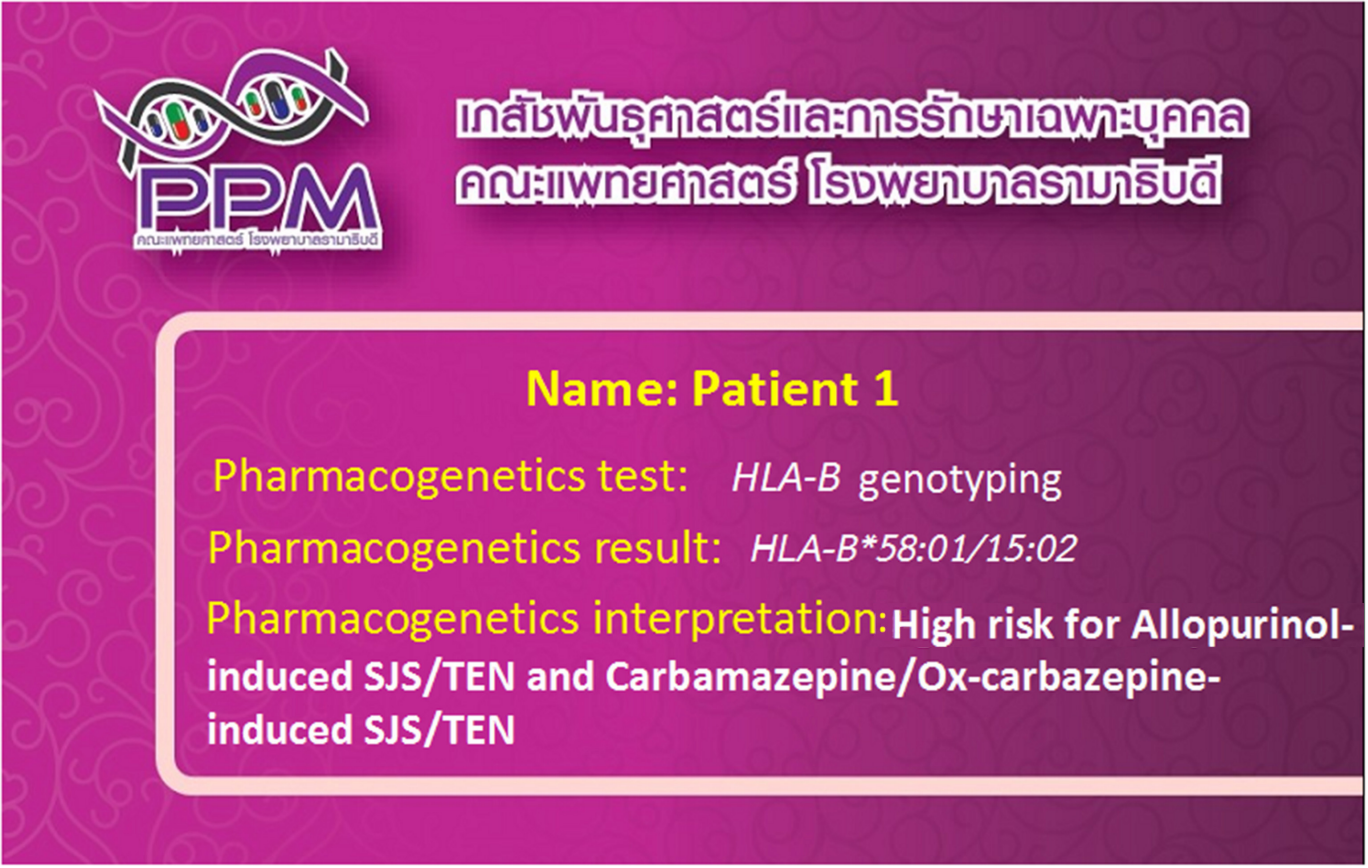
**“Pharmacogenomics cards” have been successfully implemented in clinical practice in Thailand**. The patients are tested for the HLA-B alleles which are associated with the ADRs related with the use of concerned drugs.

*HLA*-associated drug hypersensitivity is dependent upon the different populations studied, and variation in genetic backgrounds. Recently, *HLA-B* genotyping has become accessible in clinical practice, providing appropriate clinical monitoring, patient counseling and recommendations for treatment. Interestingly, “pharmacogenetic tests” and “pharmacogenomic cards” (Figure 1) have been successfully implemented in clinical practice in Thailand at the Laboratory for Pharmacogenomics, Somdech Phra Debaratana Medical Center, Ramathibodi Hospital (Sukasem et al., 2014a). Recently, HLA-B^*^15:02 screening has been preliminarily introduced by the National Health Security Office (NHSO), Thailand, to screen patients who may be at risk of carbamazepine-induced SJS/TEN.

In conclusion, our data suggest that *HLA-B* genotype should be screened before medication is prescribed to reduce the incidence of drug hypersensitivity. These findings provided useful information in the study of pharmacogenomics and *HLA-B* polymorphisms in the Thai population. The frequency of the *HLA-B* alleles associated with drug hypersensitivity will help in designing alternative treatment regimens or better therapy for affected individuals. Understanding of the mechanism underlying drug hypersensitivity reactions requires further investigation.

### Conflict of interest statement

The reviewer Wichittra Tassaneeyakul declares that, despite having collaborated with authors Apichaya Puangpetch and Chonlaphat Sukasem, the review process was handled objectively and no conflict of interest exists. The authors have no relevant affiliations or financial involvement with any organization or entity with a financial interest in or financial conflict with the subject matter or materials discussed in the manuscript.
